# Prevalence and pathogenic potential of *Shewanella* species in oysters and seawater collected from the Chesapeake Bay and Maryland Coastal Bays

**DOI:** 10.3389/fmicb.2025.1502443

**Published:** 2025-01-24

**Authors:** Tahirah Johnson, Gary P. Richards, John Jacobs, Howard Townsend, Esam Almuhaideb, Detbra Rosales, Paulinus Chigbu, Ligia Dasilva, Salina Parveen

**Affiliations:** ^1^University of Maryland Eastern Shore, Department of Agriculture, Food and Resource Science, Princess Anne, MD, United States; ^2^United States Department of Agriculture, Agricultural Research Service, Dover, DE, United States; ^3^National Oceanic and Atmospheric Administration, NOS, NCCOS, Cooperative Oxford Laboratory, Oxford, MD, United States

**Keywords:** *Shewanella* species, oysters, seawater, pathogenicity, hemolytic, Chesapeake Bay, Maryland Coastal Bay, environmental influences

## Abstract

*Shewanella* is a genus of Gram-negative marine bacteria with some species associated with human and shellfish illnesses. This study evaluated the abundance of *Shewanella* species in oysters and seawater from the Chesapeake and Maryland Coastal Bays at four sites between 2019 and 2021. Physicochemical parameters such as temperature, salinity, dissolved oxygen, turbidity, pH, chlorophyll-a, rainfall within the last 48 h, total dissolved solids, and atmospheric pressure were also recorded to evaluate if there was a correlation between environmental parameters and the level of *Shewanella.* The highest total *Shewanella* counts were 1.8 × 10^7^ CFU/g in oysters and 4.0 × 10^2^ CFU/mL in seawater. 16S rRNA sequencing was performed on 1,344 representative isolates of which 890 (713 oyster, 177 seawater) were confirmed as *Shewanella* within 16 species. The top four species isolated from oysters and seawater were *S. khirikhana* a known shrimp pathogen (49%), *S. marisflavi* (19%), *S. loihica* (11%), and *S. algae* (8%). Testing for alpha and beta hemolysis were performed on all confirmed *Shewanella* isolates. Beta hemolysis was observed in 405 (46%) of the isolates of which 313 were in oysters and 92 in seawater. In oysters, beta-hemolysis was most prevalent in *S. khirikhana* (233 of 344 isolates, 68%), while in seawater 64 of 92 isolates (70%) were beta-hemolytic strains. Beta-hemolysis suggests that these could be potentially pathogenic strains. Correlations were performed between physicochemical attributes of the seawater and *Shewanella* counts. Only seawater temperature and dissolved oxygen correlated with *Shewanella* counts (*r* = 0.45 and − 0.41), respectively. No correlations were observed between the physicochemical parameters and *Shewanella* abundances in oysters. Results suggest that virulent strains of *Shewanella* may be present in oysters and seawater from the Chesapeake and Maryland Coastal Bays, perhaps as a consequence of rising seawater temperatures.

## Introduction

*Shewanella* spp. are Gram-negative, facultatively anaerobic, singly flagellated, oxidase and catalase positive, rod-shaped bacteria that can produce H_2_S (Goyal et al., 2011). Members of the family *Shewanellaceae* are naturally occurring marine bacteria, distributed worldwide (Hau and Gralnick, 2007; Lemaire et al., 2020). Over 70 species of *Shewanellaceae* have been identified (Lemaire et al., 2020); however, there is still uncertainty of exactly how many species are truly human pathogens. Currently, five species have been associated with human infection: *S. algae*, *S. putrefaciens* (Hau and Gralnick, 2007), *S. xiamensis* (Zong, 2011), *S. haliotis* (Poovorawan et al., 2013; Zhang et al., 2018; Liu et al., 2019) and *S. upenei* (Yousfi et al., 2017). One recently identified species, *S. khirikhana,* has been associated with disease in cultured shrimp (Prachumwat et al., 2020). *Shewanella* is a genus that contains species that are human and shellfish pathogens as well as species that are widely recognized as seafood spoilage organisms.

*Shewanella* isolates can often exhibit hemolytic activity on blood agar plates, which is indicative of their ability to lyse red blood cells. Beta-hemolytic activity is a common feature associated with pathogenicity in various bacterial species. For instance, in *Staphylococcus aureus*, beta hemolysins are key virulence factors that contribute to tissue invasion and immune evasion, and their activity can be significantly reduced by inhibiting the *ClpP* protease, central regulator of virulence factors (Böttcher and Sieber, 2008). Similarly, *Shewanella* species such as *S. algae* and *S. baltica* have been shown to possess beta-hemolytic activity, and this trait may be predictive of their potential virulence, especially given their ability to grow at human body temperature (Richards et al., 2008). Moreover, the presence of genes encoding hemolysins, like those found in the *S. algae STY3* genome, further supports the potential for these species to be pathogenic under the right conditions. These genes include those similar to *hlyA*, which encodes a toxin capable of causing cell lysis, and other genes involved in a hemolysin operon (Wu et al., 2018; Tamez et al., 2020). While *S. baltica* has not been commonly reported as a cause of human infections (Myung et al., 2009), the increasing number of illnesses linked to *Shewanella* infections in the last decade underscores the importance of considering these bacteria as emerging pathogens (Ng et al., 2022). These findings highlight the need for further research to elucidate the specific virulence mechanisms in *Shewanella* and their implications for human health.

Alpha hemolysins function by partially breaking down hemoglobin into biliverdin, resulting in a characteristic green discoloration around colonies on blood agar (Tasnim, 2017). This activity can damage host cells through mechanisms such as pore formation in cell membranes, as seen with pneumolysin in *Streptococcus pneumoniae* (Tasnim, 2017), which disrupts cellular integrity and can lead to cell death. Alpha hemolysins can interfere with host cell signaling pathways, suppressing immune responses and promoting bacterial survival. For example, pneumolysin produced by *S. pneumoniae* has been shown to impair host immune cells, allowing the bacteria to colonize and spread more effectively (Tasnim, 2017). In *Shewanella*, genomic analyses suggest the presence of genes encoding toxins similar to alpha hemolysins found in other bacteria, such as RTX-like toxins (Alav et al., 2021). These RTX toxins, like pneumolysin in *S. pneumoniae*, play roles in immune evasion and persistence within the host by targeting cell membranes and disrupting immune signaling (Tasnim, 2017). In *Shewanella*, RTX-like toxins may play a similar role in damaging host tissues, contributing to bacterial survival and colonization (Tasnim, 2017; Alav et al., 2021).

Pathogenic species reported most frequently in the literature are *S. algae* and *S. putrefaciens,* however, in the past, there have been problems identifying the microbes at the species level using conventional microbiological culture and biochemical analyses (Gram et al., 1999). More recently, this problem has been resolved by incorporating 16S rRNA gene sequencing, as the DNA sequences provide an opportunity for more definitive identification (Hau and Gralnick, 2007; Zong, 2011; Liu et al., 2013). Humans can become infected from certain *Shewanella* species by exposing cuts and abrasions in the skin to contaminated seawater, and from various water activities such as swimming, fishing, and boating. The consumption of raw or undercooked seafood has also been associated with illness (Myung et al., 2009; Liu et al., 2019). Some species are pathogenic to shellfish, causing production losses in the seafood industry (Huang et al., 2018). In humans, *S. algae* can cause soft tissue infections; bone, joint, and ear infections; lower respiratory tract infection; and occasional gastroenteritis (Holt et al., 2005; Dey et al., 2015; Takata et al., 2017; Kitaoka et al., 2019). It can also cause necrotizing fasciitis and typically attacks individuals who are immunocompromised, or have underlying conditions such as renal failure, diabetes mellitus, liver disease, and malignancies (Holt et al., 2005; Dey et al., 2015; Takata et al., 2017; Kitaoka et al., 2019).

*Shewanella* spp. can be readily isolated from seafood and environmental samples (Richards et al., 2008; Kang et al., 2013; Calvert County Health Department, 2017). Potentially pathogenic species were identified in the Delaware Bay with high numbers present in oysters and seawater during the summer months (Richards et al., 2008). Kang et al. (2013) isolated antibiotic resistant *S. putrefaciens* from shellfish in the West Sea in Korea. In 2017, cases of *Shewanella* infections have also been reported around the Chesapeake Bay area (Calvert County Health Department, 2017). In addition, one case has been reported during 2018 in New York involved an 87-year-old patient diagnosed with an infection caused by *Shewanella haliotis* (Liu et al., 2019). These cases underscore the potential pathogenicity of *Shewanella* species, particularly those isolated from marine environments. Furthermore, information is limited concerning the prevalence of this bacterium in oysters and seawater from the Chesapeake Bay and the Maryland Coastal Bays.

The objectives of this study were to: (i) isolate, identify, and quantify species of *Shewanella* in the Chesapeake and Maryland Coastal Bays; (ii) determine the levels of hemolytic *Shewanella* spp. in seawater and oysters as a potential indicator of their pathogenic potential, (iii) evaluate the seasonal distribution of *Shewanella* spp. in oysters and seawater at various sampling sites, and (iv) correlate *Shewanella* levels in oyster and seawater samples with physicochemical parameters of the seawater.

## Materials and methods

### Sampling sites

**Figure 1:**
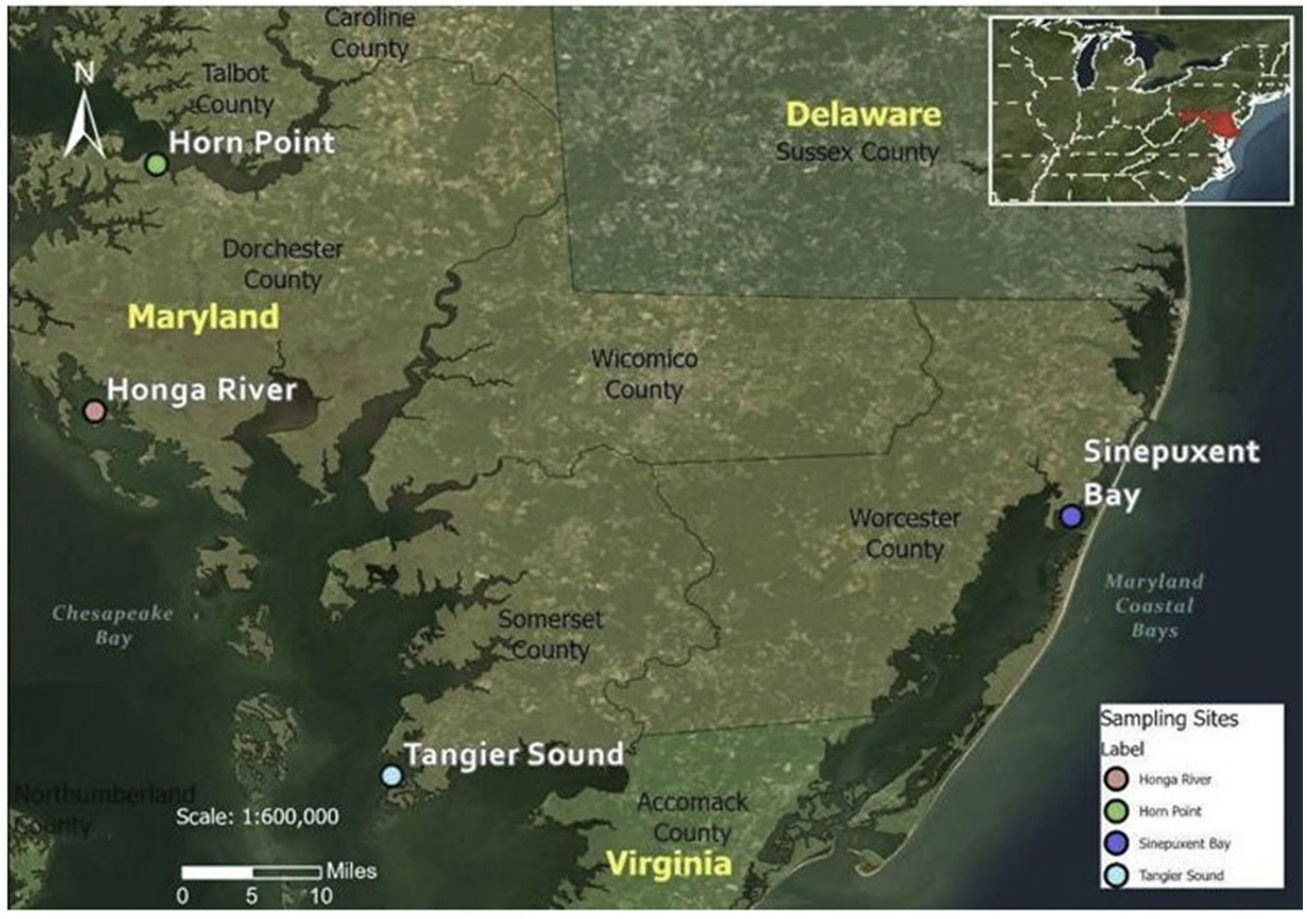
The three sample collection sites in Chesapeake Bay and one sample collection site in Maryland Coastal Bay. Coordinates for sampling sites: Horn Point (38°35′33”N 76°07′47”W); Honga River (38°20′03″N 76°11″W); Tangier Sound (37°57′09″N 75°53′01″W); Sinepuxent Bay (38°13′27″N 75°10′21″W).

Study sites were selected to represent the range of salinities where *Shewanella* spp. may be present as well as represent the optimum conditions for the survival of oysters. Three sites in the Chesapeake Bay (Horn Point, Honga River, Tangier Sound) and one site in the Maryland Coastal Bays (Sinepuxent Bay on the Atlantic Ocean) were selected based on salinities, water quality, accessibility, and availability of oysters (Figure 1). The sites in the Maryland Coastal Bays represent the higher salinity range (generally 27–35 ppt). Salinities at selected sites were representative of oyster-growing areas nationally. The Chesapeake Bay is located on the East coast of the US within the states of Maryland and Virginia. The Chesapeake Bay is the largest estuary in North America. It is 314 km (195 miles) long and between 6 and 48 km (4 and 30 miles) wide, covering an area of 8,384 km^2^ (3,237 miles^2^), with 4,470 km^2^ (1,726 miles^2^) in Maryland. Salinities increase from north to south as one moves downstream from Maryland’s portion of the Bay into Virginia where the Chesapeake Bay meets the Atlantic Ocean. The Maryland Coastal Bays encompass the Assawoman Bay, Chincoteague Bay, Isle of Wright Bay, Newport Bay, Sinepuxent Bay, and St. Martin River (Rodgers et al., 2014). For this current study, because of the scarcity of wild oysters in some locations, we utilized oysters cultured at a site in the Sinepuxent Bay.

### Collection of samples

Eighty-one oyster samplings were performed, consisting of 12 oysters per sampling, and 82 seawater samplings (1-liter each) were collected from three study sites in the Chesapeake Bay and one site along the Maryland Coastal Bays monthly from June 2019 through October 2021 (Supplementary Table S1); except for 4 months (March–June 2020) as the university was closed due to the COVID-19 lockdown. At Horn Point, a total of 22 water and 22 oyster samplings were conducted. Similarly, 21 water and 21 oyster samplings were performed in the Honga River. In Tangier Sound, a total of 23 water and 23 oyster samplings were conducted. Lastly, at Maryland Coastal Bay, 16 water and 15 oyster samplings were performed. On a few occasions, we were not able to collect samples from the Maryland Coastal Bays site due to inclement weather and technical issues. Physicochemical parameters such as salinity, water and air temperature, turbidity, total dissolved solids, dissolved oxygen, pH, chlorophyll-*a*, precipitation, and atmospheric pressure were recorded using a YSI Pro Plus Multi-parameter Meter (Yellow Springs Instrument Co., Yellow Springs, OH). Upon harvesting, oysters were bagged and chilled in a cooler filled with ice and a sheet of bubble wrap was used to prevent direct contact of samples and ice. A Smart Button data logger (TEquipment, Long Branch, NJ) was used to monitor and verify that shipping temperatures of the oysters remained at or above 10°C.

### Oysters—isolation and enumeration

Twelve oysters were divided into three sub-samples, using four oysters per subsample. The entire amount of oyster meat and liquor was homogenized in a small blender (Waring Commercial, 7010S, Wilmington, NC) for 1.5 min at high speed. Twenty-five grams of the oyster homogenate were added to a large blender (Waring Commercial, 7010S, Wilmington, NC) along with 225 mL of 0.1% peptone dilution buffer (weight/volume) and blended for 1 min at high speed. Serial dilutions of each homogenate (to a final dilution of 10^−6^) and water sample (to a final dilution of 10^−4^) were prepared in 0.1% peptone dilution buffer. *Shewanella algae* and *E. coli* were used as positive and negative controls for all experiments. One hundred microliters of each dilution were spread plated in duplicate onto Iron Agar (Condalab, Madrid, Spain) plates using an EddyJet 2 W- Spiral Plater (Neutec Group Inc., IUL S.A., Farmingdale, NY) followed by incubation of the plates for 48 h at 35°C. After incubation, the number of bacterial colonies on each plate were counted and recorded. Up to 10 (or 10% if CFU > 100) presumptive colonies were picked and streaked onto tryptic soy agar (TSA) (Difco™, Becton, Dickinson and Company, Sparks, MD) with 0.5% added NaCl (1% total NaCl) and incubated over night to ensure sufficient growth and isolation. The Bactidrop Oxidase Test Kit (Remel, Lenexa, KS) was used to determine if the isolates were oxidase positive since *Shewanella* are oxidase positive. Only oxidase positive colonies were inoculated into tryptic soy broth (TSB) (Difco™, Becton, Dickinson and Company, Sparks, MD) with 1% added NaCl (1.5% total NaCl) and incubated overnight at 35°C to create fresh culture. Glycerol stocks were prepared by combining equal volumes of the fresh culture and 50% sterile glycerol and storage at −80°C for subsequent hemolysis testing and 16S rRNA sequencing.

### Seawater—isolation and enumeration

Seawater was divided into three sub-samples and was used to make 10-fold serial dilutions from 10^0^ to 10^−4^ in 0.1% peptone buffer. One hundred microliters of the seawater dilutions as well as the undiluted seawater was spread plated onto iron agar using the EddyJet Spiral Plater. These plates were then incubated for 48 h at 35°C. After incubation, the number of bacterial colonies on each plate was counted and recorded. Up to 10 (or 10% if CFU > 100) presumptive colonies were picked and streaked for oxidase and hemolysis testing, as described above. Glycerol stocks were prepared from colonies on the TSA plates.

### Testing the hemolysis

For hemolysis testing, the isolates were evaluated for alpha, or beta hemolytic activity on the TSA with 5% sheep’s blood agar plates. After isolates were incubated overnight on TSA with 1% NaCl, a 3-mm inoculum loop was used to pick a single colony from the TSA plate. This colony was mixed into TSB with 1% added NaCl and incubated overnight at 35°C to create a fresh culture. The fresh culture was then streaked onto TSA with 5% sheep’s blood agar plates (Thermo Fisher Scientific, Waltham, MA), and was incubated overnight at 35°C. Hemolytic activity was assessed the next day: alpha hemolysis was identified by dark green discoloration (indicative of partial hemoglobin breakdown), beta hemolysis by neon green zones of complete hemolysis, and gamma hemolysis by the absence of hemolytic activity.

### 16S rRNA sequencing

16S rRNA sequencing was employed for the identification of presumptive *Shewanella* isolates at Genewiz (2019–2020) and Azenta (2021) (South Plainfield, NJ). Genomic DNA was extracted from overnight cultures of the isolates grown in 1% tryptic soy agar using a DNeasy tissue kit (Qiagen, Valencia, CA). PCR amplification of a fragment of the 16S rRNA gene was performed on each genomic preparation using forward primer 63F (5-CAG GCC TAA CAC ATG CAA GTC-3) and reverse primer 1387R (5-GGG CGG WGT GTA CAA GGC-3), along with master mix and Taq DNA polymerase, following the protocol described by Marchesi et al. (1998). Sequencing was carried out in both forward and reverse directions using the 63F and 1387R primers, respectively, provided by SeqXcel Inc. (San Diego, CA) resulting in approximately 750 bp of sequence data in each direction. The sequences were viewed in Chromas (version 2.6.6, Technelysium Ply Ltd., South Brisbane, Australia) and trimmed. Identification was carried out by comparing the sequences to the GenBank database using the BLASTn program (Richards et al., 2008; Altschul et al., 1990). The sequences were submitted to NCBI/GenBank by EzBiome Inc. (Gaithersburg, MD). Finally, the presumptive *Shewanella* colonies were quantified by multiplying the percentage of isolates confirmed to be *Shewanella* via 16S rRNA sequencing by the total number of presumptive colonies, with the data converted to CFU/g or CFU/mL of sample.

### Statistical analysis

Pearson’s correlations (Cor, RStudio 2022.07.1 + 554; R Studio Team, Vienna, Austria) were performed to assess similarities between CFU levels and physicochemical parameters. Correlations were considered weak when the *r* values were between 0.40 and 0.49, moderate when *r* values were between 0.50 and 0.69, and strong when *r-*values were ≥ 0.70. One-way ANOVA tests were used to assess differences between sampling sites, sampling months, and sampling years, with significance defined as *p*-value <0.05. Levene’s test was used to assess variances among oyster and water samples, with significance defined as *p-*value <0.05.

## Results

### Identification of *Shewanella* species

**Figure 2:**
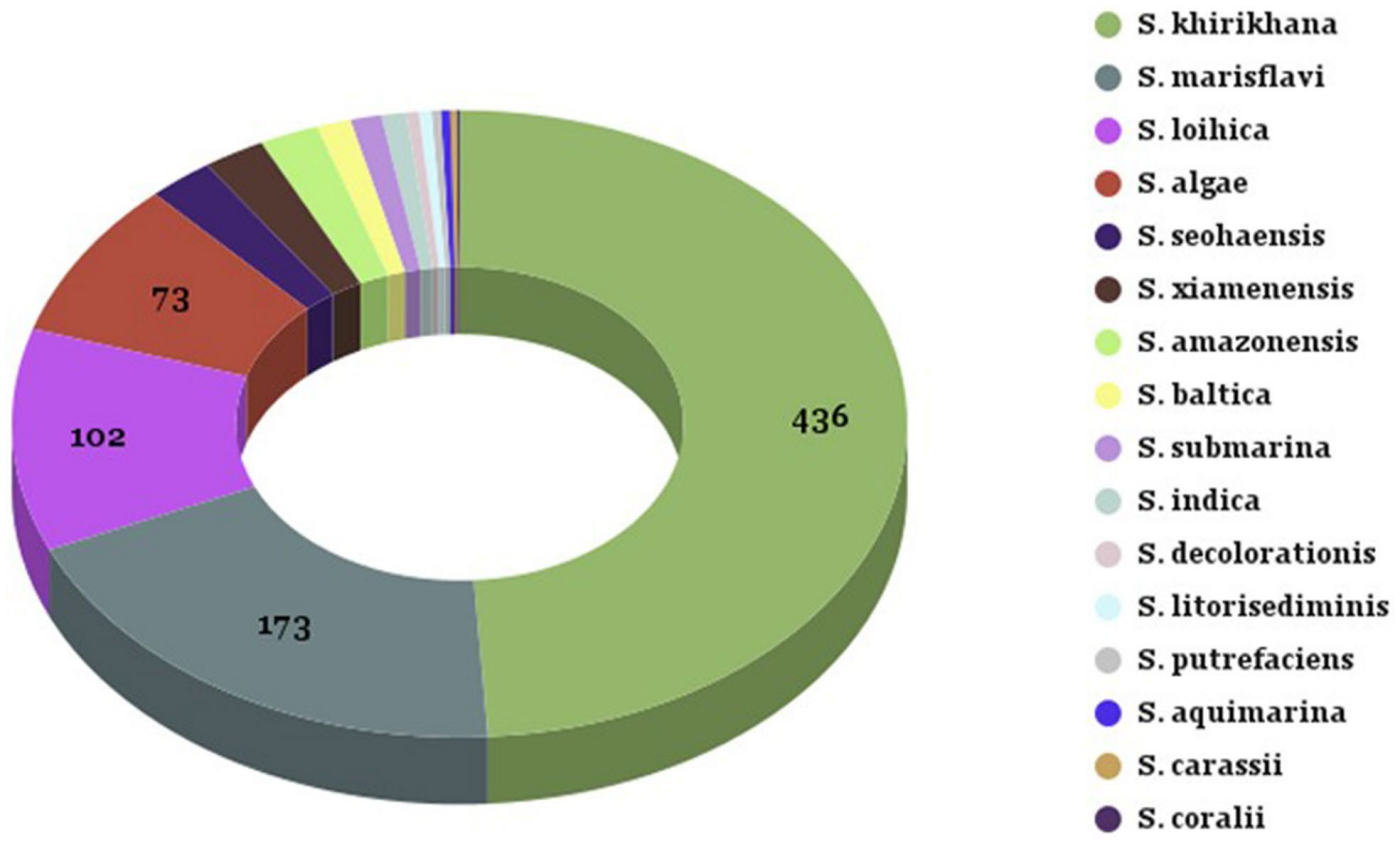
Summary of *Shewanella* species identified from oyster and seawater samples. Summary of *Shewanella* species identified from oyster and seawater samples collected from Chesapeake and Maryland Coastal Bays. The legend on the right corresponds to the color-coded sections of the pie chart, representing the different *Shewanella* species and their respective counts.

Out of 1,344 (1,099 oyster, and 245 seawater) presumptive *Shewanella* isolates, a total of 713 and 177 isolates were confirmed in oysters and seawater, respectively, using 16S rRNA sequencing. Sixteen different *Shewanella* spp. were identified, with three species that contain strains that are human pathogens (*S. algae, S. xiamensis,* and *S. putrefaciens*). The highest abundances of species detected in seawater and oysters were 436 *S. khirikhana*, 173 *S. marisflavi*, 102 *S. loihica*, and 73 *S. algae* (Table 1; Figure 2). These four species constitute 88% of the total *Shewanella* identified. Three species known to cause human illness were identified: *S. algae* (8%), *S. xiamensis* (2%), and *S. putrefaciens* (0.3%). Comparing the Chesapeake Bay and the Maryland Coastal Bays led to a higher frequency of isolating *S. khirikhana* in the Chesapeake Bay (54%), while over 60% of the Maryland Coastal Bays’ isolates were identified as *S. marisflavi* or *S. loihica.*

**Table 1:** Frequency and hemolytic activity of *Shewanella* species identified in oyster (OY) and seawater (SW) samples.

*Shewanella* species identified	No. and % total isolates^a^		No. of isolates by sample type		No. and % of beta (*β*) and alpha (*α*) hemolytic isolates^a^							
	#	%	OY	SW	#β		%β		# α		%α	
					OY	SW	OY	SW	OY	SW	OY	SW
*S. khirikhana*	436	49%	344	92	233	64	68%	70%	111	28	32%	30%
*S. marisflavi*	173	19%	146	27	27	6	19%	22%	119	21	82%	78%
*S. loihica*	102	11%	91	11	19	3	21%	27%	72	8	79%	73%
*S. algae*	73	8%	49	24	10	16	20%	67%	39	8	80%	33%
*S. seohaensis*	21	2%	18	3	3	0	17%	0%	15	3	83%	100%
*S. xiamenensis*	20	2%	12	8	3	1	25%	13%	9	7	75%	88%
*S. amazonensis*	19	2%	17	2	10	1	59%	50%	7	1	41%	50%
*S. baltica*	11	<1%	11	0	4	0	36%	0%	7	0	64%	0%
*S. submarina*	10	1%	4	6	0	1	0%	17%	4	5	100%	83%
*S. indica*	8	<1%	5	3	0	0	0%	0%	5	3	100%	100%
*S. decolorationis*	4	<1%	3	1	0	0	0%	0%	3	1	100%	100%
*S. litorisediminis*	4	<1%	4	0	1	0	25%	0%	3	0	75%	0%
*S. putrefaciens*	3	<1%	3	0	2	0	67%	0%	1	0	33%	0%
*S. aquimarina*	3	<1%	3	0	0	0	0%	0%	3	0	100%	0%
*S. carassii*	2	<1%	2	0	1	0	50%	0%	1	0	50%	0%
*S. coralii*	1	<1%	1	0	0	0	0%	0%	1	0	100%	0%
Totals:	890	95%^b^	713	177	313	92	35%	10%	400	84	45%	9%

OY, oyster samples; SW, seawater samples; α, alpha hemolytic; β, beta hemolytic.

a Percentages 1% were rounded to the nearest whole number.

b The total percentage is less than 100% due to rounding.

### Identification of potentially pathogenic (hemolytic) *Shewanella*

Beta hemolysis was detected in 313 oyster isolates and 92 seawater isolates together representing 45% of the total confirmed *Shewanella* isolates from both sample types combined (Table 1). The majority were *S. khirikhana* with 233 out of a total of 436 beta hemolytic isolates (53%) from oysters and 64 out of 92 beta hemolytic isolates (70%) from seawater. Ten oyster isolates of *S. algae* (20%) were beta hemolytic while 16 beta hemolytic strains were observed in seawater isolates (67%) (Table 1). Alpha hemolysis was detected in 400 oyster isolates and 84 seawater isolates together representing 54% of the total confirmed *Shewanella* isolates from both sample types combined (Table 1). The majority were *S. marisflavi* with 119 out of a total of 400 alpha hemolytic isolates (30%) from oysters and 21 out of 84 alpha hemolytic isolates (25%) from seawater. Alpha hemolysis was also present in ≈ 30% of *S. khirikhana* isolates from oysters and seawater (Table 1). Thirty-nine oyster isolates of *S. algae* (80%) were alpha hemolytic while 8 alpha hemolytic strains were observed in seawater isolates (33%) (Table 1). It is unclear whether the pathogenicity of *S. khirikhana* toward shrimp is related to the presence of either alpha or beta hemolysis. Interestingly, *S. marisflavi* showed the highest number of alpha hemolytic strains in oyster samples, but a low number of beta hemolytic strains (Table 1).

### Regional distribution of hemolytic strains

The regional distribution of hemolytic strains of *Shewanella* was analyzed over a three-year period (Table 2). All the isolates were either alpha or beta hemolytic or both. The number of beta hemolytic isolates in oysters from the four sites are as follows: Horn Point (160 isolates), Honga River (105 isolates), Tangier Sound (38 isolates), and the Maryland Coastal Bays (10 isolates) (Table 2). The beta hemolytic isolates obtained from seawater were Horn Point (53 isolates), followed by Tangier Sound (26 isolates), and Honga River (13 isolates). No isolates were reported from the Maryland Coastal Bay, perhaps due to the lower number of samples analyzed from that site. The number of alpha hemolytic isolates in oysters from the four sites were as follows: Horn Point (119 isolates), Honga River (96 isolates), Tangier Sound (134 isolates), and the Maryland Coastal Bays (61 isolates) (Table 2). The alpha hemolytic isolates obtained from seawater were Horn Point (39 isolates), Honga River (7 isolates), Tangier Sound (30 isolates), and the Maryland Coastal Bays (8 isolates) (Table 2). The percentage of total beta hemolytic isolates and alpha hemolytic isolates from oysters for each of the four sites are as follows: Horn Point (57% beta/43% alpha), Honga River (52% beta/48% alpha), Tangier Sound (23% beta/77% alpha), and Maryland Coastal Bays (14% beta/86% alpha). The Horn Point site contained the highest number and percentage of beta hemolytic isolates, and the Tangier Sound site contained the highest number of alpha hemolytic isolates; suggesting that these sites may have more strains potentially pathogenic to fish, shellfish, or humans. Similar comparisons performed for seawater revealed the following: Horn Point (58% beta/42% alpha), Honga River (65% beta/35% alpha), Tangier Sound (46% beta/54% alpha), and the Maryland Coastal Bays (0% beta/100% alpha).

**Table 2:** Total number of alpha and beta hemolytic *Shewanella* strains isolated from oysters and seawater at four sites over 3 years.

Site	2019				2020				2021				Totals			
	OY		SW		OY		SW		OY		SW		OY		SW	
	α	β	α	β	α	β	α	β	α	β	α	β	α	β	α	β
HP	41	61	16	25	45	60	13	23	33	39	10	5	119	160	39	53
HR	55	33	0	8	16	37	4	4	25	35	3	1	96	105	7	13
TA	86	31	20	24	26	4	3	1	12	3	7	1	124	38	30	26
MD	56	10	3	0	4	0	0	0	1	0	5	0	61	10	8	0
Totals	238	135	39	57	91	101	20	28	71	77	25	7	400	313	84	92

OY, oyster samples; SW, seawater samples; α, alpha hemolytic; β, beta hemolytic; HP, Horn Point; HR, Honga River; TA, Tangier Sound; MD, Maryland Coastal Bays.

### Prevalence and seasonal distribution of total *Shewanella*

**Figure 3:**
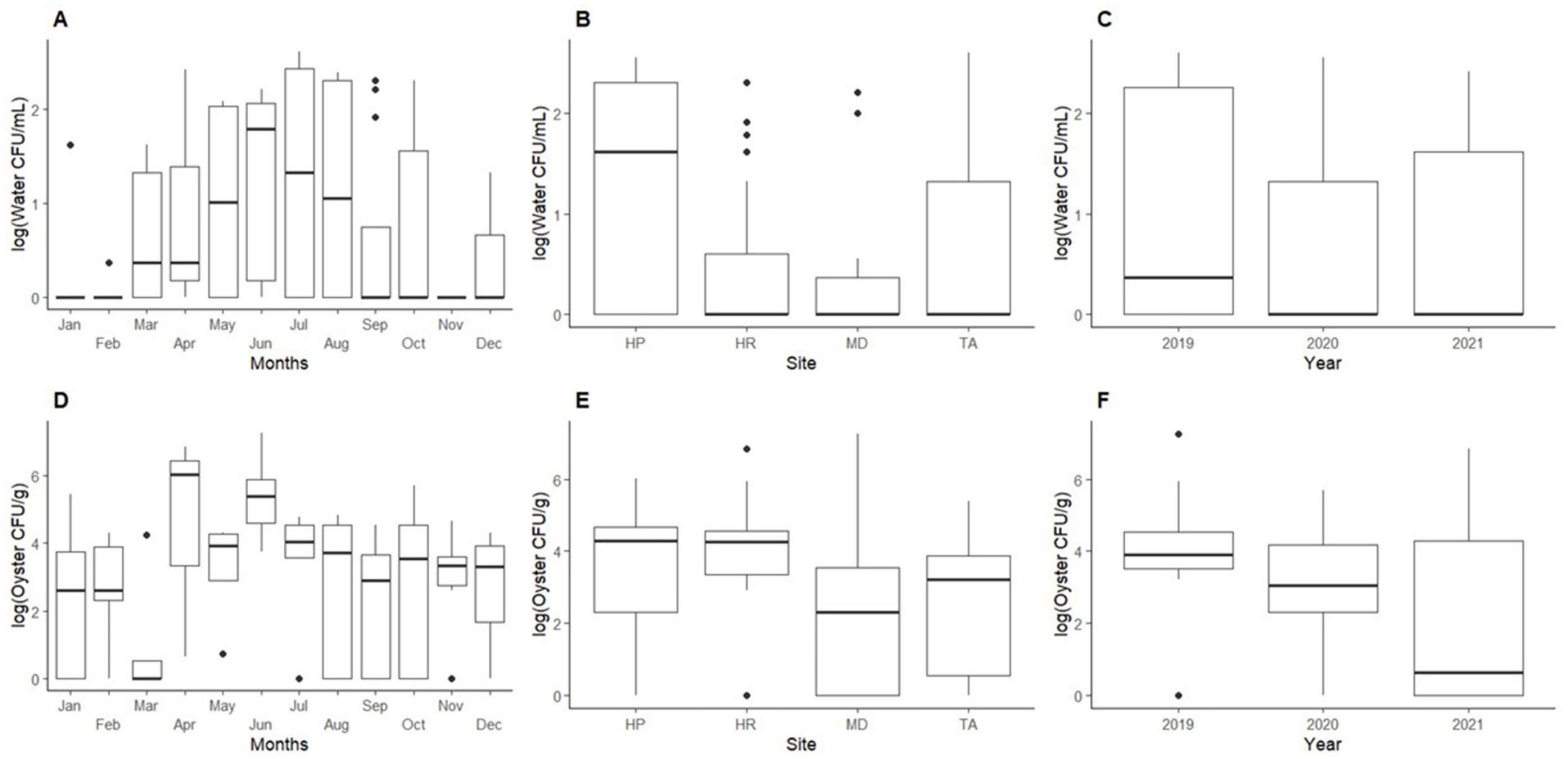
Mean abundance of *Shewanella* found in oyster and seawater. Top graphs show abundances of *Shewanella* in seawater by: **(A)** month, **(B)** sampling site, and **(C)** year. Bottom graphs show abundances of *Shewanella* in oysters by: **(D)** month, **(E)** sampling site, and **(F)** year. Abbreviations for graphs **(B,E)**: HP, Horn Point; HR, Honga River; TA, Tangier Sound; and MD, Maryland Coastal Bay.

ANOVA tests were performed for *Shewanella* counts in seawater and oyster samples compared to sampling month, sampling site, and sampling year. For *Shewanella* counts in oyster samples, there were no significant differences between sampling site (*p =* 0.214), sampling month (*p =* 0.295), or sampling year (*p =* 0.280). For seawater, significant differences in *Shewanella* abundances were observed for sampling site (*p <* 0.01), and sampling month (*p <* 0.05), while sampling year showed no significant differences (*p* = 0.132) (Figure 3).

In water samples, 71% of the total isolates sequenced were identified as *Shewanella*. The highest counts of *Shewanella* were observed in May through August (Figure 3A). Horn Point had higher *Shewanella* occurrences from June through September with counts ranging from undetectable to 3.60 × 10^2^ CFU/mL (July 2020). For the same period, Honga River, Tangier Sound, and Maryland Coastal Bay had counts from undetectable to 2.00 × 10^2^ CFU/mL (August 2019), undetectable to 4.00 × 10^2^ CFU/mL (July 2019); and undetectable to 2.60 × 10^1^ CFU/mL (October 2019), respectively.

In oyster samples, 65% of the sequenced isolates were identified as *Shewanella.* The highest detection for oysters was observed from April through June (Figure 3D), oyster counts ranged from undetectable to 7.20 × 10^5^ CFU/g at Horn Point (June 2021); undetectable to 8.40 × 10^5^ CFU/g at Honga River (June 2019); undetectable to 2.40 × 10^5^ CFU/g at Tangier Sound (June 2021); and undetectable to 1.80 × 10^7^ CFU/g at Maryland Coastal Bay (June 2019) (Figure 3E).

### Environmental influence on *Shewanella*

Seawater physiochemical parameters at the time of sample collections are given in Table 3. The seawater temperature ranged from 17.8–19.6°C with Horn Point averaging the lowest temperature and Maryland Coastal Bays averaging the highest water temperature. Salinity ranged from 12 to 31 ppt with Maryland Coastal Bays averaging the highest salinity levels and Horn Point averaging the lowest. Dissolved oxygen ranged from 7.5–8.6 mg/L with Tangier Sound averaging the highest levels and Maryland Coastal Bays averaging the lowest. Turbidity ranged from 5.8–10.6 Formazin Nephelometric Unit (FNU) with Horn Point and Honga River averaging the highest levels and Tangier Sound averaging the lowest. The pH was ≈ 8 for all sampling sites. Chlorophyll-a ranged from 6 to 8 μg/L with Horn Point averaging the highest measurements and Maryland Coastal Bay with the lowest. Precipitation ranged from about 3.0 to 7.4 mm with Horn Point receiving the least amount of average rainfall within 48 h prior to sampling and Tangier Sound averaging the most. Total dissolved solids ranged from 1,319 to 2,976 mg/L with Maryland Coastal Bay averaging the highest and Horn Point averaging the lowest. Atmospheric pressure consistently measured 765 mm Hg at all sampling sites (Table 3). Physicochemical water parameters such as water temperature, turbidity, and pH had lower averages in 2020 compared to 2019 and 2021.

**Table 3:** Average physiochemical parameters by sampling site and year.

	Sampling site				Sampling year		
	HP	HR	TA	MD	2019	2020	2021
Temp (°C)	17.8	18.10	17.55	19.62	19.41	16.30	18.70
Sal (ppt)	12.12	15.10	17.64	30.86	19.51	18.01	16.99
DO (mg/L)	8.01	8.20	8.60	7.53	7.98	8.57	8.14
Turb (FNU)	10.57	10.59	5.82	7.48	11.22	6.67	8.50
pH	7.80	7.70	7.96	7.92	8.10	7.90	7.77
Chlo-a (μg/L)	8.33	7.36	6.57	5.78	6.66	6.62	7.13
Precip (mm)	3.05	3.81	7.37	5.08	8.64	5.08	2.54
TDS (mg/L)	1,319	1,597	1856	2,976	2001	1841	1776
Baro (mm Hg)	764	765	765	765	765	766	764

HP, Horn Point; HR, Honga River; TA, Tangier Sound; MD, Maryland Coastal Bays. The parameters include seawater temperature (Temp °C), salinity (Sal ppt), dissolved oxygen (DO mg/L), turbidity (Turb FNU), pH level of the water (pH), chlorophyll-α concentration (Chlo-α μg/L), total precipitation within the 48 h prior to sample collection (Precip mm), total dissolved solids (TDS mg/L), and barometric pressure (Baro mm Hg).

*Shewanella* spp. were consistently isolated at higher concentrations in oysters compared to seawater (*p <* 0.05). This trend may be attributed to the filter-feeding behavior of *Crassostrea virginica*. Correlation coefficients (*r*-value) for various physicochemical parameters and total *Shewanella* abundances are shown in Table 4. For seawater, temperature had a low positive correlation (*r =* 0.45) with *Shewanella* abundance, while dissolved oxygen showed a low negative correlation (*r =* −0.41). For oysters, there were no notable correlations between the various parameters and abundances (*r ≤* 0.35).

**Table 4:** Pearson correlation coefficients comparing the physiochemical parameters of the harvest water and oysters with the abundance of *Shewanella* isolation.

	Correlation coefficient (r value)	
Physiochemical parameter	Seawater (CFU/mL)	Oysters (CFU/g)
Water temperature	**0.45** ^**a**^	0.35
Salinity	−0.24	−0.30
Dissolved oxygen	**−0.41** ^**a**^	−0.13
Turbidity	0.27	0.27
Ph	0	0.18
Chlorophyll-a	−0.17	−0.07
Precipitation	−0.08	0.03
Total dissolved solids	−0.20	−0.24
Atmospheric pressure	0.07	0.24

a Correlations reflect comparisons between surface water parameters and CFU counts for both water and oysters. Signifies moderate correlation. All other correlations are weak except for *r* = 0, which reflects no correlation.

## Discussion

This is the first comprehensive study that investigates the presence, diversity, influence of environmental parameters, and pathogenic potential of *Shewanella* species in oysters and seawater samples recovered from the Chesapeake Bay and the Maryland Coastal Bay in the Mid-Atlantic region of the USA. To maximize the range of collection conditions encountered during the study, low and high salinity oyster harvesting sites were selected. Compared to seawater, oyster samples contained more *Shewanella* at all sites. This was expected due to the ability of oysters to bioaccumulate large numbers of microorganisms as they filter the water (Center for Disease Control and Prevention, 2022). Our results are consistent with the finding of a previous study that reported higher levels of *Photobacterium damselae* and *Shewanella* in oysters in comparison to seawater (Richards et al., 2008). The diversity of *Shewanella* species identified in this study was unexpected and some of these species have not been previously reported in U.S. coastal waters. It is uncertain whether their presence was influenced by changes in climate, better identification methods, or the fact that *Shewanella* research has been very limited. Nearly half of the isolates identified in this study were *S. khirikhana,* which was only recently recommended as a new species (Prachumwat et al., 2020). It has a 16S rRNA sequence similar to that of *S. amazonensis. S. khirikhana* was associated with disease of cultivated shrimp in Thailand (Prachumwat et al., 2020). We were unable to identify previous reports of *S. khirikhana* or *S. amazonensis* species in the U.S. It is unclear whether *S. khirikhana* is responsible for disease in shrimp, or other shellfish, or fish species in U.S. aquaculture or in wild caught seafoods. Surveillance for this shellfish pathogen may be prudent because of its high prevalence in mid-Atlantic bays and the high numbers of alpha and beta hemolytic strains detected.

While the levels of total *Shewanella* varied among the bays, similar results were observed in the levels of total *Shewanella* between sample types (oyster and seawater) and the three sampling sites in the Chesapeake Bay. Although dissolved oxygen, chlorophyll-a levels, and seawater pH showed some correlation with *Shewanella* levels in seawater, no correlation was observed between the physicochemical parameters and *Shewanella* abundances in oysters. There can be many factors outside of environmental influences (Abirami et al., 2021; Marsay et al., 2023) that can lead to higher abundances of *Shewanella* and other bacteria. This can include host factors (Lemaire et al., 2020), nutrient availability, biofilm formation, genetic adaptations (Lemaire et al., 2020), anthropogenic influences, and enhanced metabolic activity of bacteria (Abirami et al., 2021).

The distribution of *Shewanella* species may be influenced by various factors, including environmental conditions and geographical location (Marsay et al., 2023). Different regions exhibit unique sets of physical and chemical parameters that shape the microbial communities thriving within them (Hanson et al., 2019). Specific environmental conditions, such as temperature, salinity, oxygen availability, and nutrient availability can shape the prevalence and adaptation of many microbial communities (Hanson et al., 2019; Marsay et al., 2023). The presence of geographical barriers, such as ocean currents or continental landmasses, can restrict the dispersal of certain species, resulting in regional variations in species composition (Hanson et al., 2019).

By comparing the Chesapeake Bay and the Maryland Coastal Bays, it was observed that isolation of *S. khirikhana* in the Chesapeake Bay was high, while over 90% of the Maryland Coastal Bays’ isolates were identified as *S. marisflavi* or *S. loihica.* Additionally, other studies have reported the isolation of several other *Shewanella* species, namely *S. algae* (Gram et al., 1999; Richards et al., 2008; Taherzadeh et al., 2014; Zago et al., 2020), *S. putrefaciens* (Richards et al., 2008; Kang and So, 2016), and *S. baltica* (Richards et al., 2008; Zago et al., 2020), which were also found in the Chesapeake Bay. These *Shewanella* species have been discussed and documented in studies conducted in the Delaware Bay (Richards et al., 2008), Danish Coastal waters (Gram et al., 1999), Persian Gulf (Taherzadeh et al., 2014), Adriatic Sea (Zago et al., 2020) and the West Sea (Kang and So, 2016).

Although all *Shewanella* isolates in this study were either alpha or beta hemolytic, the pathogenic potential of *Shewanella* species is not yet defined. If oysters become contaminated with hemolytic *Shewanella*, there could be potential safety concerns for shellfish consumers. The bacteria could cause health problems such as gastrointestinal illness or even more serious conditions like sepsis, particularly in individuals with weakened immune systems. Literature suggests that bacteria containing hemolysins may have higher antibiotic resistance and be more pathogenic toward humans (Jarraud et al., 2002; Nizet, 2002; Schroeder et al., 2017; Motamedi et al., 2018). We found that 45% of *Shewanella* isolates in this study had beta hemolytic activity. For example, *S. algae* is documented as pathogenic toward humans and exhibited a high percentage of beta-hemolytic activity in this study. With 45% of *Shewanella* isolates demonstrating beta hemolytic activity, including 20% of *S. algae* isolates from oysters and seawater, this finding raises concerns about potential pathogenicity.

*S. algae* has been increasingly recognized as an emerging human pathogen capable of causing serious infections, including those of the skin, soft tissue, bloodstream, and peritoneum (Kitaoka et al., 2019; Tamez et al., 2020). The presence of putative hemolysin genes in *S. algae* as discussed by Tamez et al. (2020), provides a molecular basis for its hemolytic activity and pathogenicity. Enzymes that are responsible for *β*-hemolysis have been established as a virulence factor for a variety of bacterial pathogens such as *Enterococci* (Nizet, 2002), *Escherichia coli* (Bhakdi et al., 1988), *Listeria monocytogenes* (Goebel et al., 1988; Raybourne, 2002), and *Vibrio parahaemolyticus* (Okuda and Nishibuchi, 1998). The presence of hemolytic activity in *Shewanella* has significant ramifications for the safety and quality of oysters and may well serve as an indicator of virulence in *Shewanella* spp. (Richards et al., 2008).

*Shewanella algae* demonstrates remarkable environmental adaptability and potential pathogenicity, as evidenced by its prevalence in marine ecosystems. Our findings on the prevalence and hemolytic activity of *S. algae* can be contextualized by comparing them with the study by Tseng et al. (2018). While Tseng et al. (2018) reported that *S. algae* was present in approximately 23% of shellfish samples and 28% of water samples, our study observed a higher prevalence with 67% in oysters and 33% in seawater. This difference may be attributed to geographical variations and/or sampling methods. However, the high prevalence suggests that oysters frequently harbor this bacterium, though its direct impact on oyster health has not been discussed. Notably, Tseng et al. (2018) examined hemolytic activity at both 25 and 37°C, finding that *S. algae* exhibited stronger hemolytic activity at human body temperature with 100% hemolysis after 72 h at 37°C compared to 50% at 25°C. In contrast, our study maintained a constant temperature of 35°C for all hemolytic testing and found beta hemolytic activity to be 20% in oysters and 67% in seawater, while alpha hemolytic activity was drastically higher in oysters at 80%, compared to 33% in seawater. Furthermore, Tseng et al. (2018) findings on *S. algae’s* adaptability to varying temperatures and salinities are comparable to our results; we noted a moderate correlation (*r* = 0.45) between water temperature and the abundance of *Shewanella* in our seawater samples (as noted in Table 4). This adaptability allows *S. algae* to survive in various marine environments, including oyster habitats, potentially affecting oyster microbiomes. These comparisons underscore the pathogenic potential of *S. algae* in both marine environments and human hosts, emphasizing the importance of continued monitoring for food safety implications.

A study by Richards et al. (2008) evaluated oysters and seawater from the Delaware Bay for the presence of potentially pathogenic *Shewanella* and *Photobacterium* species using the API 20E System (biMerieux Industries, Hazelwood, MO), which is a biochemical kit used to evaluate primarily human pathogens for clinical diagnoses. They found that out of 1,421 isolates tested, 12% (*n* = 170) of all picked colonies were identified as presumptive *S. putrefaciens*. The only *Shewanella* listed in the API database was *S. putrefaciens* of which some were subsequently identified as *S. algae.* 16S rRNA sequencing was performed and revealed a host of different *Shewanella* species, not present in the API database (Richards et al., 2008). Differences in assay methodologies between the Delaware study and the present study at the Maryland sites prevent direct comparison of results. Advances in scientific techniques and technologies have allowed researchers to uncover and distinguish between previously unknown or misidentified species. As a result, the number of recognized *Shewanella* species has expanded over time.

The levels of total *Shewanella* in the Chesapeake and Maryland Coastal Bays can provide important information about the potential risks associated with consuming seafood from those areas. However, it is important to note that the presence of *Shewanella* alone does not necessarily indicate a safety concern, as not all species of *Shewanella* are pathogenic and for those that are, the levels of bacteria alone may not be sufficient to cause illness. *Shewanella* had the highest levels observed in 2019, while the least amount was observed in 2021. In 2019–2021, *S. khirikhana* was the most frequently isolated species from oyster and seawater samples. It is unclear if *S. khirikhana* outcompetes other species or if water conditions play some role in enhancing their growth or reproduction over other species. There was no significant variation between the years for the four most frequently isolated *Shewanella* species (*S. khirikhana, S. marisflavi, S. loihica,* and *S. algae*).

Knowledge is limited on the extent of disease caused by *Shewanella* species from the Chesapeake Bay and Maryland Coastal Bay or the origins of infections, such as from the consumption of contaminated seafood, by cuts or abrasions in the skin after exposure to a contaminated marine environment, or by recreational water activities. The detection of potentially pathogenic species in this study suggests the need for continued monitoring of *Shewanella* occurrences and further investigation of any association this bacterium may have with disease. Studies on factors that correlate with abundances of *Shewanella* to physicochemical parameters should be continued and expanded to other coastal regions. Monitoring the levels of pathogenic species should be continued, and investigations on antibiotic susceptibility should be performed. Enhanced reporting of *Shewanella* cases should be adopted to ascertain more accurate data on disease prevalence and its association with seafood consumption, particularly during the warmer months when *Shewanella* levels are the highest in both seawater and oysters.

However, the limitations of this study include limited sample size from the Maryland Coastal Bays due to logistical challenges, such as inclement weather and boating issues. In addition, the COVID-19 lockdown in 2020 caused the omission of critical sampling months (March–June). Furthermore, while hemolytic activity was assessed to infer pathogenic potential, direct evidence linking *Shewanella* species to seafood-related illnesses was not evaluated. Lastly, while correlations with physicochemical parameters were explored, the potential role of other environmental factors, such as pollution or nutrient inputs, remains to be evaluated, limited the number of samples collected from Maryland Coastal Bays, which may have impacted regional comparisons. Addressing these limitations in future studies will enhance our understanding of the ecological and clinical significance of *Shewanella* species in estuarine environments.

## Conclusion

*Shewanella* species are of significant interest due to their pathogenic potential to humans and marine organisms, with multiple transmission routes including marine water, seafood, and wound infections. This study is the first comprehensive investigation into the diversity and pathogenic potential of *Shewanella* species recovered from oysters and seawater in the Chesapeake Bay and Maryland Coastal Bays. By filling critical data gaps on the abundance, diversity, and pathogenic potential of *Shewanella*, this research raises awareness of this emerging pathogen and lays the groundwork for developing intervention technologies to prevent or mitigate *Shewanella*-associated illnesses. Continued research in this area will be crucial to better understand the environmental factors driving the emergence of these pathogens and for informing public health strategies.

## Data Availability

The datasets presented in this study can be found in online repositories. The names of the repository/repositories and accession number(s) can be found at: https://www.ncbi.nlm.nih.gov/genbank/, OR941802; https://www.ncbi.nlm.nih.gov/genbank/, OR943574.

## Author’s note

The use of trade names or commercial products in this publication is solely for the purpose of providing specific information and does not imply recommendation or endorsement by the U.S. Department of Agriculture (USDA). The USDA is an equal opportunity provider and employer.
