# Supplementary material for: Prevalence and pathogenic potential of Shewanella species in oysters and seawater collected from the Chesapeake Bay and Maryland Coastal Bays
Source: Front Microbiol. 2025 Jan 24;16:1502443. doi: 10.3389/fmicb.2025.1502443 (PMC11802537; doi:10.3389/fmicb.2025.1502443)
Supplement: Supplementary file 1 [file Data_Sheet_1.pdf]

Supplementary Table 1: Summary of Seawater Sample Collection

Sample Date	Sample Location	Quantity of water samples collected[a]	Quantity of oyster samples collected[b]
6/4/2019	Maryland Coastal Bay	1 (1L)	12
7/15/2019	Maryland Coastal Bay	1 (1L)	12
8/12/2019	Maryland Coastal Bay	1 (1L)	12
9/17/2019	Maryland Coastal Bay	1 (1L)	12
10/23/2019	Maryland Coastal Bay	1 (1L)	12
11/11/2019	Maryland Coastal Bay	1 (1L)	12
12/26/2019	Maryland Coastal Bay	1 (1L)	12
1/31/2020	Maryland Coastal Bay	1 (1L)	12
2/20/2020	Maryland Coastal Bay	1 (1L)	12
7/22/2020	Maryland Coastal Bay	1 (1L)	12
8/1/2020	Maryland Coastal Bay	1 (1L)	12
9/17/2020	Maryland Coastal Bay	1 (1L)	12
1/11/2021	Maryland Coastal Bay	1 (1L)	12
5/27/2021	Maryland Coastal Bay	1 (1L)	12
6/17/2021	Maryland Coastal Bay	1 (1L)	12
7/27/2021	Maryland Coastal Bay	1 (1L)	0
8/31/2021	Maryland Coastal Bay	1 (1L)	0
6/10/2019	Honga River	1 (1L)	12
7/22/2019	Honga River	1 (1L)	12
8/19/2019	Honga River	1 (1L)	12
9/23/2019	Honga River	1 (1L)	12
10/21/2019	Honga River	1 (1L)	12
11/4/2019	Honga River	1 (1L)	12
12/9/2019	Honga River	1 (1L)	12
1/13/2020	Honga River	1 (1L)	12
2/3/2020	Honga River	1 (1L)	12
7/21/2020	Honga River	1 (1L)	12
8/3/2020	Honga River	1 (1L)	12
9/14/2020	Honga River	1 (1L)	12
10/10/2020	Honga River	1 (1L)	12
12/3/2020	Honga River	1 (1L)	12
1/21/2021	Honga River	1 (1L)	12
2/15/2021	Honga River	1 (1L)	12
3/23/2021	Honga River	1 (1L)	12
4/27/2021	Honga River	1 (1L)	12
5/20/2021	Honga River	1 (1L)	12
6/14/2021	Honga River	1 (1L)	12
7/13/2021	Honga River	1 (1L)	12
7/8/2019	Horn Point	1 (1L)	12
8/5/2019	Horn Point	1 (1L)	12
9/9/2019	Horn Point	1 (1L)	12

10/7/2019	Horn Point	1 (1L)	12
11/4/2019	Horn Point	1 (1L)	12
12/9/2019	Horn Point	1 (1L)	12
1/13/2020	Horn Point	1 (1L)	12
2/3/2020	Horn Point	1 (1L)	12
3/19/2020	Horn Point	1 (1L)	12
7/21/2020	Horn Point	1 (1L)	12
8/3/2020	Horn Point	1 (1L)	12
9/14/2020	Horn Point	1 (1L)	12
10/10/2020	Horn Point	1 (1L)	12
11/17/2020	Horn Point	1 (1L)	12
12/29/2020	Horn Point	1 (1L)	12
01/21/2021	Horn Point	1 (1L)	12
02/15/2021	Horn Point	1 (1L)	12
03/05/2021	Horn Point	1 (1L)	12
04/27/2021	Horn Point	1 (1L)	12
05/18/2021	Horn Point	1 (1L)	12
06/14/2021	Horn Point	1 (1L)	12
07/13/2021	Horn Point	1 (1L)	12
6/25/2019	Tangier Sound	1 (1L)	12
7/31/2019	Tangier Sound	1 (1L)	12
8/27/2019	Tangier Sound	1 (1L)	12
9/19/2019	Tangier Sound	1 (1L)	12
10/28/2019	Tangier Sound	1 (1L)	12
11/19/2019	Tangier Sound	1 (1L)	12
12/12/2019	Tangier Sound	1 (1L)	12
1/22/2020	Tangier Sound	1 (1L)	12
2/12/2020	Tangier Sound	1 (1L)	12
3/24/2020	Tangier Sound	1 (1L)	12
7/27/2020	Tangier Sound	1 (1L)	12
8/9/2020	Tangier Sound	1 (1L)	12
9/24/2020	Tangier Sound	1 (1L)	12
10/20/2020	Tangier Sound	1 (1L)	12
11/9/2020	Tangier Sound	1 (1L)	12
12/7/2020	Tangier Sound	1 (1L)	12
01/22/2021	Tangier Sound	1 (1L)	12
02/09/2021	Tangier Sound	1 (1L)	12
03/25/2021	Tangier Sound	1 (1L)	12
04/14/2021	Tangier Sound	1 (1L)	12
05/19/2021	Tangier Sound	1 (1L)	12
06/06/2021	Tangier Sound	1 (1L)	12
07/13/2021	Tangier Sound	1 (1L)	12

[a] Each seawater sample corresponds to a single 1L bottle of seawater collected, which was

subsequently divided into three subsamples for analysis. For further details, refer to the "Seawater – Isolation and Enumeration" subsection in the Materials and Methods section.

[b]Each oyster sample represents 12 oysters collected per sampling event, which were divided into three subsamples, each containing four oysters. For additional information, refer to the "Oyster – Isolation and Enumeration" subsection in the Materials and Methods section.
